# Morroniside ameliorates inflammatory skeletal muscle atrophy *via* inhibiting canonical and non-canonical NF-κB and regulating protein synthesis/degradation

**DOI:** 10.3389/fphar.2022.1056460

**Published:** 2022-12-23

**Authors:** Xiangjiao Yi, Jianguo Tao, Yu Qian, Feng Feng, Xueqin Hu, Taotao Xu, Hongting Jin, Hongfeng Ruan, Hou-Feng Zheng, Peijian Tong

**Affiliations:** ^1^ The First Affiliated Hospital of Zhejiang Chinese Medical University (Zhejiang Provincial Hospital of Traditional Chinese Medicine), Hangzhou, Zhejiang, China; ^2^ Diseases & Population (DaP) Geninfo Lab, School of Life Sciences, Westlake University, Hangzhou, Zhejiang, China; ^3^ Westlake Laboratory of Life Sciences and Biomedicine, Hangzhou, Zhejiang, China; ^4^ Institute of Basic Medical Sciences, Westlake Institute for Advanced Study, Hangzhou, Zhejiang, China; ^5^ The First Affiliated Hospital of Anhui University of Chinese Medicine, Hefei, China; ^6^ College of Life Sciences, Zhejiang University, Hangzhou, China

**Keywords:** morroniside, muscle atrophy, denervation, inflammation, autophagylysosomal pathway, ubiquitin-proteasome system, protein synthesis and degradation, genetic association study

## Abstract

No drug options exist for skeletal muscle atrophy in clinical, which poses a huge socio-economic burden, making development on drug interventions a general wellbeing need. Patients with a variety of pathologic conditions associated with skeletal muscle atrophy have systemically elevated inflammatory factors. Morroniside, derived from medicinal herb *Cornus officinalis*, possesses anti-inflammatory effect. However, whether and how morroniside combat muscle atrophy remain unknown. Here, we identified crucial genetic associations between TNFα/NF-κB pathway and grip strength based on population using 377,807 European participants from the United Kingdom Biobank dataset. Denervation increased TNFα in atrophying skeletal muscles, which inhibited myotube formation *in vitro*. Notably, morroniside treatment rescued TNFα-induced myotube atrophy *in vitro* and impeded skeletal muscle atrophy *in vivo*, resulting in increased body/muscles weights, No. of satellite cells, size of type IIA, IIX and IIB myofibers, and percentage of type IIA myofibers in denervated mice. Mechanistically, *in vitro* and/or *in vivo* studies demonstrated that morroniside could not only inhibit canonical and non-canonical NF-κB, inflammatory mediators (IL6, IL-1b, CRP, NIRP3, PTGS2, TNFα), but also down-regulate protein degradation signals (Follistatin, Myostatin, ALK4/5/7, Smad7/3), ubiquitin-proteasome molecules (FoxO3, Atrogin-1, MuRF1), autophagy-lysosomal molecules (Bnip3, LC3A, and LC3B), while promoting protein synthesis signals (IGF-1/IGF-1R/IRS-1/PI3K/Akt, and BMP14/BMPR2/ALK2/3/Smad5/9). Moreover, morroniside had no obvious liver and kidney toxicity. This human genetic, cells and mice pathological evidence indicates that morroniside is an efficacious and safe inflammatory muscle atrophy treatment and suggests its translational potential on muscle wasting.

## 1 Introduction

Skeletal muscle atrophy is characterized by the decrease in muscle mass, strength and/or physical performance, increasing the likelihood of adverse outcomes, including frailty, fall-related fractures, physical disability, social exclusion, hospitalization, ultimately increased health care costs and mortality (Lin et al., 2022). No drugs are currently approved to counter muscle atrophy (Otzel et al., 2021). Physical activity and nutritional supplementation are the two major interventions actively implemented in clinical settings, but the effects are limited and the compliance is poor (Chhetri et al., 2018).

Various conditions, such as denervation (Reza et al., 2017), disuse, aging, rheumatoid arthritis (RA) (Li et al., 2020), Crohn’s disease, and cachexia, can cause muscle atrophy (Nikawa et al., 2021), which are all characterized by elevated levels of circulating pro-inflammatory mediators in patients, such as TNFα, IL-1β, and/or IL-6 (Roubenoff et al., 1994; Rosales-Antequera et al., 2022). During aging and in adult TNF-Tg mice, increased levels of TNFα induce NF-κB activation, which led to loss of Pax7+ satellite cells and of MyHC IIA + myofibers (Li et al., 2020). Constitutive activation of canonical NF-κB signaling in satellite cells attenuates skeletal muscle regeneration following injury in adult mice (Straughn et al., 2019). Stimulation of non-canonical NF-κB impairs myogenic differentiation, muscle stem cell function and regeneration of skeletal muscle in mice (Schmidt et al., 2021). Therefore, inflammation could play an important role in muscle atrophy. However, in human, there is a paucity of genetic studies to support the association of *Tnfα* gene and NF-κB signaling pathway in response to TNFα with muscle atrophy or decreased grip strength.

In skeletal muscle, atrophy occurs when proteolysis accelerates and/or protein synthesis reduces. Ubiquitin-proteasome and autophagy-lysosome systems are the two protein degradation systems. Ubiquitin-proteasome is the system that proteins are tagged by ubiquitin and are later selectively proteolyzed by proteasome. Previously, we and others have demonstrated that muscle-specific E3 ubiquitin ligases, atrophy gene-1/muscle atrophy F-box (Atrogin1/MAFbx) and muscle ring-finger protein 1 (MuRF1), are up-regulated by TNFα-induced NF-κB activation, and increased downstream inflammatory cytokines such as TNF-α, IL-1β or NLR Family pyrin domain containing 3 (NLRP3), which promote myosin heavy-chain (MyHC) degradation (Li et al., 2008; Huang et al., 2017; Li et al., 2020). Autophagy is another major intracellular degradation system that uses acidic lysosomal hydrolases to degrade proteins and organelles. BCL2-interacting protein 3 (BNIP3) activation promotes LC3-I conjugation with phosphatidylethanolamine to form LC3-II, which is recruited to autophagosomal membranes to elongate and form complete double-layered vesicle, the autophagosome (Fritzen et al., 2016). Moreover, the transcription factor forkhead box O type 3 (FoxO3) can not only up-regulate ubiquitin-proteasome system (Atrogin1 and MuRF1), but also activate autophagy-lysosome system (BNIP3 and LC3) (Mirzoev, 2020).

Insulin-like growth factor 1 (IGF-1)/type 1 IGF receptor (IGF-1R) and Myostatin/activin/BMP pathways impinge both on protein synthesis and breakdown (Sartori et al., 2021). IGF-1 ligand binds to IGF-1R, which can activate the intracellular adaptor protein insulin receptor substrate-1 (IRS-1) and further induce downstream PI3K/Akt to promote protein synthesis (Sartori et al., 2021). In addition, FoxO transcription factors can be down-regulated by IGF-1 treatment or Akt overexpression, which causes inhibition of ubiquitin-proteasome and autophagy-lysosome pathway (Sandri et al., 2004; Mammucari et al., 2007; Sartori et al., 2021). Myostatin/activin/BMP pathways can be divided into BMP pathway and myostatin/activin pathway based on the opposite function. BMP ligands such as BMP14 bind BMP type II receptors like BMPR2 and recruit type I receptors such as ALK2/3, which activate transcription factors Smad1/5/9(8) to form a complex with Smad4 for promoting protein synthesis (Sartori et al., 2013). However, myostatin/activin and GDF11 bind to type II receptors like ActRIIB/IIA and recruit type I receptors ALK4/5/7, which activate transcription factors Smad2/3 to form a complex with Smad4 for enhancing ubiquitination-associated protein breakdown (Sartori et al., 2009; Winbanks et al., 2012). Moreover, Smad2/3 can negatively modulate Akt and mTOR to promote protein degradation and inhibit protein synthesis, while Smad1/5/9(8) can positively regulate mTOR to promote protein synthesis (Sartori et al., 2009; Winbanks et al., 2012). Myostatin/activin ligands are inhibited extracellularly by the cytokine Follistatin (Winbanks et al., 2012). Smad6 and 7 negatively regulate the BMP and Myostatin/activin pathway in cytosol, respectively (Winbanks et al., 2016; Sartori et al., 2021).

Morroniside, a kind of iridoid glycosides, is the major active ingredient in *Cornus officinalis* (*C. officinalis*) Sieb. et Zucc. (Shanzhuyu) which is an herb used in traditional Chinese medicine (TCM) (Zhao et al., 2022). Preparations of this herb show a tonic effect and are widely used to treat liver, kidney and reproductive system diseases since ancient times (Gao et al., 2021). It has been reported that morroniside has a variety of biological effects, such as neuroprotective, antinociceptive, cardioprotective, bone-protective, and diabetes-related hepatoprotective and kidney-protective effects, as well as anti-inflammatory, anti-oxidative, anti-apoptotic activities (Gao et al., 2021). Recently we reported that morroniside attenuates apoptosis and pyroptosis of chondrocytes and ameliorates osteoarthritic development by inhibiting canonical NF-κB p65/RelA signaling (Yu et al., 2021). However, its curative activity on inflammation-associated skeletal muscle atrophy has not yet been reported.

In this study, we performed genetic association studies, functional annotation and pathway-based weighted genetic risk score using the data of 377,807 European participants from the United Kingdom Biobank dataset to examine the association of *Tnfα* gene and genetically determined NF-κB signaling in response to TNFα (including 198 genes) with grip strength. Both denervation-induced muscle atrophy mouse model and TNFα-treated C2C12 cells/primary myogenic cells were used to investigate TNFα levels in atrophying muscles and its inhibition on myotube formation *in vitro*. Notably, we investigated the potential efficacy, underlying mechanism, and side effects of morroniside in TNFα-induced C2C12 myotubes atrophy *in vitro* and/or inflammatory skeletal muscle atrophy caused by denervation *in vivo*, thus, providing an important theoretical basis for the translational application of morroniside in clinical to treat patients with inflammation-associated muscle atrophy.

## 2 Materials and methods

### 2.1 Population analyses

#### 2.1.1 Data source

The individual-level phenotype and genomic datasets were obtained from United Kingdom Biobank (Application 41376), which collected almost 500,000 participants. The information of these participants on more than 2000 traits, including sex, age, and body mass index (BMI), was documented by touch-screen questionnaires, and physical measurements (Bycroft et al., 2018). Right-hand grip strength, the outcome of this study, is measured using a Jamar J00105 hydraulic hand dynamometer (Field ID 47). After excluding non-European participants (Field ID 47) and related participants, a total of 377,807 participants with phenotype information (i.e, sex, age, BMI, and right-hand grip strength) and genotype datasets (i.e., the rate of missing genotypes was less than 10%) were included in the following analyses.

#### 2.1.2 Genetic association study for *Tnfα* gene

To explore the associations of single nucleotide polymorphisms (SNPs) within the *Tnfα* gene region with grip strength, we conducted genetic association studies. We extracted the SNPs in the region of 500 kb upstream and downstream of the *Tnfα* gene. We then excluded SNPs with a minor allele frequency <0.1% (--maf 0.001) and *p*-value for Hardy–Weinberg equilibrium ≤1.0 × 10^–6^ (--hwe 1e-06), leaving 7711 SNPs in the genetic association study. The associations of remained SNPs with right-hand grip strength were adjusted for sex, age, BMI, and the top five principal components. The novel loci was defined as the SNPs within this loci (lead SNP ±500 kb) that have not been previously reported to be associated with grip strength at a genome-wide significant level, based on the searching on GWAScatalog (19 June 2022).

#### 2.1.3 Functional annotation

We then annotated the regulatory function, including enhancers and histone modification sites, for SNPs with genome-wide significant association for right-hand grip strength (*p*-value <5 × 10-8). Specifically, the enhancer dataset was obtained from Human Enhancer Disease Database (Wang et al., 2018). For histone modifications datasets, we obtained from Encyclopedia of DNA elements, which were derived by ChIP-seq in human skeletal muscle myoblasts and multinucleated myotubes. A total of 14 markers (Ctcf, H2az, H3k4me1, H3k4me2, H3k4me3, H3k9ac, H3k9me3, H3k27ac, H3k27me3, H3k36me3, H3k79me2, H4k20me1, H3k09me3, and Ezh239875) were assessed.

#### 2.1.4 Pathway-based genetic association study

To investigate the association of NF-κB pathway in response to TNFα with right-hand grip strength, we performed the pathway-based genetic association analysis. Specifically, we used the Molecular Signature Database (MSigDB) of Gene Set Enrichment Analysis (GSEA) web tool (https://software.broadinstitute.org/gsea/index.jsp, accessed on 4 May 2022) (Liberzon, 2014) to find the genes regulated by NF-κB in response to TNFα (Supplementary Table S4). All SNPs located in the original region of these genes (based on NCBI 37.3) were collected. After genotype quality control (--maf 0.001, --hwe 1e-06), we estimated the association of remained SNPs with right-hand grip strength. We then selected the independent SNPs (--clump-p1 0.05, --clump-r2 0.1, --clump-kb 250) for the NF-κB pathway in response to TNFα. After generating the weighted genetic risk score, the association of this genetic risk score with right-hand grip strength was assessed using linear regression. To assess the robustness of the association between genetically determined NF-κB pathway in response to TNFα and right-hand grip strength, we re-selected SNPs using the different thresholds of *p*-value (i.e., 0.5, 0.4, 0.3, 0.2, and 0.1) for the association between SNPs with grip strength.

#### 2.1.5 Pathway enrichment analyses

To investigate the potential pathway that might be affected by *Tnfα* genes, we conducted pathway enrichment analysis using KOBAS online database (Bu et al., 2021). Accounting for multiple tests, a false discovery rate (FDR)-adjusted *p*-value of 0.05 was considered statistical significance.

### 2.2 *In vivo* and *in vitro* studies

#### 2.2.1 Animals

All animal experimental procedures were approved by the Institutional Animal Care and Use Committee (IACUC) of Westlake University. C57BL/6 mice obtained from Laboratory Animal Resources Center (LARC) were housed in the vivarium under specific pathogen-free conditions. 2-month-old male C57BL/6 mice were randomized and grouped according to body weight and were anesthetized through i.p. injection of Avertin at 250–500 mg/kg of body weight. The hair on the hindlimb was shaved using an electric shaver. A small incision was made and the sciatic nerve of both legs was isolated and a 5 mm nerve was cut using surgical scissors. The incision was closed with sutures. Mice received 5 mg/kg Meloxicam s.c. once daily for up to 3 days to control pain. In the sham group, the mice were subjected to similar surgical procedures but without sciatic nerve transection. To study the effect of morroniside (Chengdu Must Bio-Technology Co., Ltd, CAS No. 25406-64-8, catalog A0349), mice were i.p.-injected with vehicle (saline, Sham and Den group) or low-dose morroniside (10 μg/kg/d, Den + Mor-L group) or high-dose morroniside (20 μg/kg/d, Den + Mor-H group) respectively, starting 1 day after operation for 12 cycles. The doses of morroniside were optimized based on the literature (Liu et al., 2021; Yu et al., 2021) and our preliminary experiment. Thereafter, mice were sacrificed by euthanasia for blood, various skeletal muscle tissues, liver, and kidney harvesting for further experiments. TA muscle tissues were dehydrated in 30% sucrose in PBS for 24 h and were embedded in OCT (Sakura Finetek, catalog 4583), frozen in dry ice-cooled isopentane, and stored at −80°C for subsequent sectioning and staining.

#### 2.2.2 Cell survival and induction of myotube formation

C2C12 cells were treated with different concentrations of morroniside (0–640 μg/ml) for 48 h, then the cell survival was examined by CCK8 kit (Vazyme, catalog A311-02). Primary myogenic cells (CD45^−^; CD31^−^; CD11b^−^; Sca1^−^) were isolated according to our previously published method (Li et al., 2020). Isolated myogenic cells (CD45^−^CD11b^−^CD31^−^Sca1^−^) or C2C12 cells (a gift from Prof. Yiting Zhou, Zhejiang University) were seeded in growth medium (10% FBS, 1% Pen/Strep, 1% Glu in DMEM) until cell confluency reached ∼70%. The cells were induced with differentiation medium (2% horse serum,1%Pen/Strep, 1% Glu in DMEM) with 5, 10, or 50 ng/ml TNFα with or without lower dose of morroniside (Mor-L, 80 μg/ml) or higher dose of morroniside (Mor-H, 160 μg/ml) for 8–72 h, and the differentiation medium with TNFα and/or morroniside was changed every 12–48 h.

#### 2.2.3 Quantitative real-time PCR

Muscles were homogenized in TRIzol reagent (Invitrogen, Thermo Fisher Scientific) by Tissuelyser II (Qiagen, United States). Total RNA from muscle tissue homogenate or C2C12 myotubes was extracted using TRIzol reagent. cDNAs were synthesized using an HiScript II cDNA Synthesis Kit (Vazyme, catalog R222-01). Quantitative RT-PCR amplification was performed in a Jena Qtower384G machine using ChamQ Universal SYBR qPCR Master Mix (Vazyme, catalog Q711-02). Each sample was prepared in triplicate, and each experiment was repeated at least 3 times. Gene expression fold change was calculated using the 2^-(∆∆Ct) method, normalized against the expression of *Gapdh*.

#### 2.2.4 Western blot analysis and co-immunoprecipitation

Gastrocnemius muscles were homogenized in protein lysis buffer (1x RIPA lysis buffer (Millipore, catalog 20-188) with 5 mM NEM, 1 mM DTT, 1 mM PMSF and protease inhibitor (Roche, catalog 11836170001) using the Tissue Lyser II instrument (Qiagen, United States) at 30 Hz. For *in vitro* experiments, C2C12 cells were resuspended in protein lysis buffer. both muscle homogenates and C2C12 cells were shaken on ice for 40 min and were centrifuged at 13,300 r.p.m. for 15 min at 4°C. Protein concentration quantification was done using BCA protein assay kit (Thermofisher, catalog 23225). Whole-cell lysates (10–30 μg protein/lane) were loaded in 4%-15% SDS-PAGE gels and transferred to PVDF membranes. Immunoblotting was carried out using antibodies to TNFα (1:1000, Cell Signaling, catalog 11948T), RelA (1:1000, Cell Signaling, catalog 8242), p50 (1:1000, Cell Signaling, catalog 13586S), RelB (1:1000, Cell Signaling, catalog 10544S), p52 (1:500, Santa Crus, catalog sc-7386), MyHC (1:500, R&D, catalog MAB4470) and GAPDH (1:3000, Goodhere, catalog AB-P-R 001). For ubiquitination assay, 500 μg of protein lysates with 103 nM Ubiquitin-Aldehyde (South Bay Bio, CA, United States, catalog SBB-PS0031) were incubated with MyHC antibody (1:500, R&D, catalog MAB4470), and precipitated proteins were Western-blotted using an Ub antibody (santa cruz, catalog sc-8017). Bands were visualized using ECL chemiluminescence (Thermo Fisher, catalog 34577).

### Immunofluorescence staining

Cryosections (10 μm thick) were fixed with 4% PFA for 10 min, washed and blocked with 0.2% Triton-100 and 10% normal goat serum in PBST for 30 min at RT and with 3% affinipure Fab fragment anti-mouse IgG (H + L) and anti-mouse IgM (Jackson Immuno Research, West Grove, PA, United States, catalog 115-007-003 and 115-006-020) for 1 h at RT. Primary antibodies to MyHC-IIA (1:40, DSHB, Iowa City, IA, United States, catalog SC-71), MyHC-IIB (1:40, DSHB, catalog BF-F3), Pax7 (1:40, DSHB, catalog Pax7), laminin (1:1000, Sigma-Aldrich, St. Louis, MO, United States, catalog L9393) were incubated at 4 °C overnight. On the second day, Alexa Fluor 568/488-conjugated Goat anti-Mouse IgG1/IgM Secondary Antibody (1:400, Invitrogen, Carlsbad, CA, United States, A21124/A21042) was incubated for 1 h at RT. For primary myogenic cells and C2C12 cells differentiated myotube staining, primary antibody to MyHC (1:100, R&D Systems, Minneapolis, MN, United States, catalog MAB4470) was incubated at 4°C overnight. On the second day, Alexa Fluor 488-conjugated goat anti-mouse IgG (1:200, Invitrogen, catalog A11001) was incubated at RT for 1 h. The stained slides were mounted with VECTASHIELD Antifade Mounting Medium with or without DAPI (Vector Laboratories, Burlingame, CA, United States, catalog H-1200-10 or H-1000-10) and imaged using an Olympus (Shinjuku, Japan) FV3000 inverted Confocal Laser Scanning Microscope. The stained plates were imaged using Olympus (Shinjuku, Japan) IX83 inverted Motorized Fluorescence Microscope.

#### 2.2.5 Serum alanine aminotransferase (ALT), aspartate aminotransferase (AST), urea nitrogen and creatinine quantification

Mice were sacrificed by euthanasia and blood was drawn from the heart with a 1 ml syringe. The serum was separated for measurement of serum ALT (catalog #C009-2-1), serum AST (catalog #C010-2-1), serum urea nitrogen (catalog #C013-2-1) and serum creatinine (catalog #C011-2-1) according to the manufacturer’s instructions (Nanjing Jiancheng Institute of Bioengineering, China) and previous description (Jin et al., 2017).

#### 2.2.6 Hematoxylin and eosin (HE) staining

Liver was cut into 0.5 cm wide pieces and kidney was cut into two identical pieces along the coronal axis, and fixed in 10% buffered formalin for 1–2 days at 4°C. Samples were dehydrated in a series of gradient ethanol and xylene solutions and embedded in paraffin. 4 μm thick sections were stained with HE according to previous description (Yi et al., 2021).

### 2.3 Statistical analysis

Population statistical analysis has been described above. In experiment studies, all results are given as the mean ± SD. Statistical analysis was performed using GraphPad Prism 8.0.1 software (GraphPad Software Inc., San Diego, CA, United States). Unpaired, 2-tailed Student’s *t* test was used for comparisons between 2 groups. One-way ANOVA and Turkey’s multiple comparisons test were used for comparisons among 3 or more groups. Two-way ANOVA and Tukey’s multiple comparisons test were used for comparisons among multiple groups of two factors. *p* values less than 0.05 were considered statistically significant.

## 3 Results

### 3.1 Population genetic evidence reveals *Tnfα* gene as a candidate gene for grip strength

**FIGURE 1:**
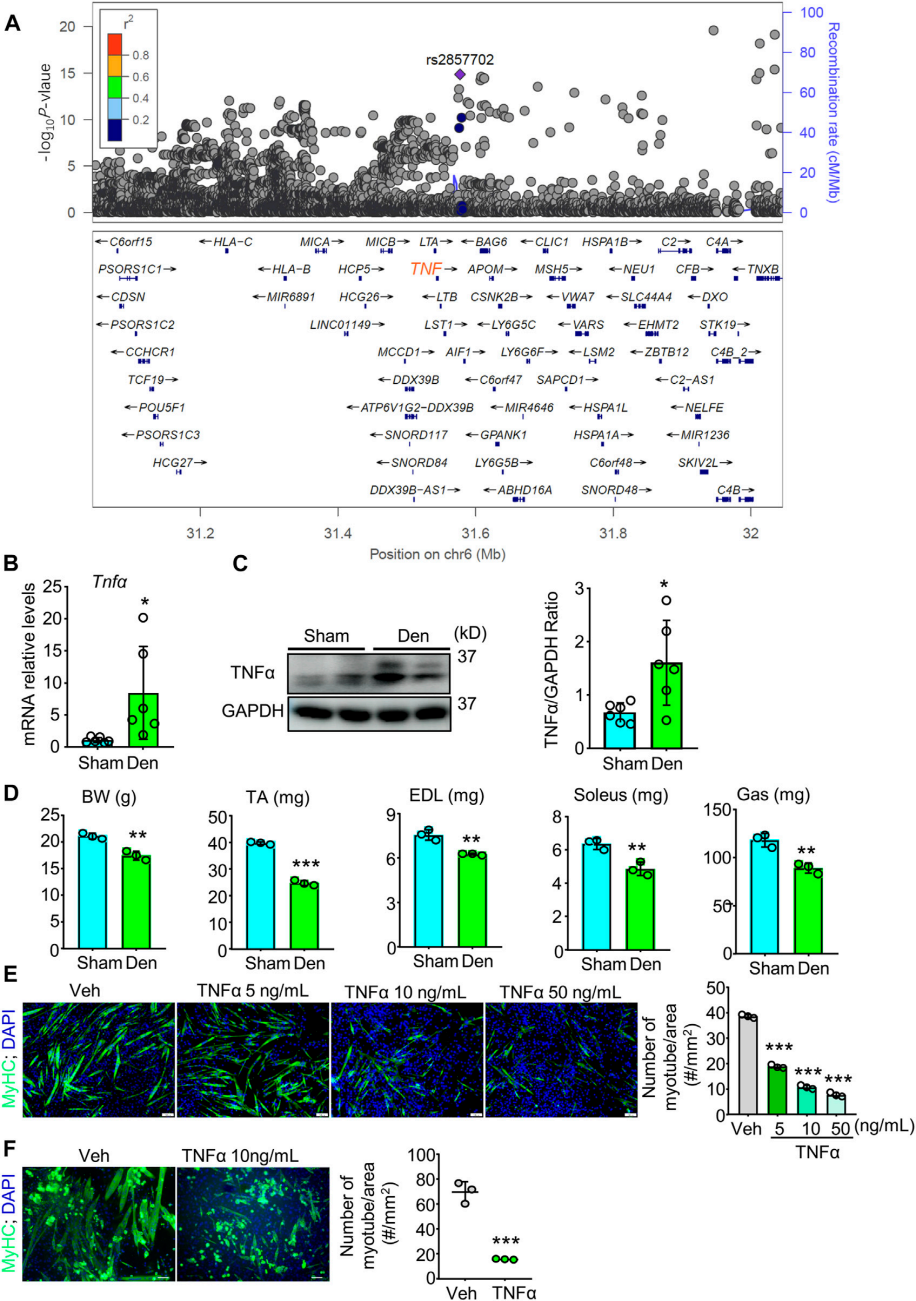
TNFα, associated with grip strength in population, increases in atrophied muscle induced by denervation and induces muscle atrophy *in vivo* and *in vitro*. **(A)** Genetic association study analysis for grip strength to find SNPs with genome-wide significant association for right-hand grip strength. There was a novel loci, and the SNP rs2857702 (*p*-value = 1.411e-15), represented with purple rhombus, was genome-wide significantly associated with right-hand grip strength and was close to *TNF* (synonyms*: Tnfα*) gene. **(B–D)** 2-month-old male C57BL/6 sham and denervated (Den) mice were harvested on day 12 after surgery. n = 3–6/group. **(B–C)** TNFα mRNA levels **(B)** and protein levels **(C)** in gastrocnemius muscles. **(D)** body weight (BW), Lean weights of the tibialis anterior (TA), extensor digitorum longus (EDL), soleus, and gastrocnemius (Gas) muscles. **(E–F)** Effects of indicated different concentrations of TNFα on myotube formation from C2C12 cells **(E)** and primary myogenic cells (MCs; CD45^−^; CD31^−^; CD11b^−^; Sca1^−^) **(F)** isolated from skeletal muscles of 3-month-old C57BL/6 mice. No. of MyHC-positive myotube/area was measured. n = 3/group; *p < 0.05, **p < 0.01; ***p < 0.001.

TNFα is suggested to be involved in many muscle atrophy animal models including denervation (Li et al., 2020; Yamauchi et al., 2021). To investigate if *Tnfα* gene is associated with muscle atrophy or decreased grip strength in human, we performed a genetic association study to find candidate SNPs associated with grip strength, a gold marker for muscle function, in European population using 377,807 European participants from the United Kingdom Biobank dataset. Interestingly, within the *Tnfα* gene region, we identified a novel loci and found 341 SNPs that were genome-wide significantly associated with right-hand grip strength (Supplementary Table S1; Figure 1A). 317 of these genome-wide significant SNPs were located in the region of enhancer and/or 14 histone modification markers in human skeletal muscle myoblasts or multinucleated myotubes (Supplementary Table S2), suggesting *Tnfα* gene as a candidate gene for muscle atrophy and grip strength in population.

### 3.2 TNFα increases in atrophying skeletal muscles induced by denervation and induces myotube atrophy *in vitro*

To examine the role of TNFα in denervation-induced muscle atrophy, we removed a part of sciatic nerve for adult C57BL/6 mice and compared TNFα levels in gastrocnemius muscles from sham and denervated mice. We found that the mean mRNA and protein levels of TNFα were higher in samples from denervated mice than from sham mice (Figures 1B,C). We next compared the skeletal muscle phenotypes of sham and denervated mice and found that body weight (BW), the lean mass of tibialis anterior (TA), extensor digitorum longus (EDL), soleus, and gastrocnemius muscles were lower in the denervated mice (Figure 1D). To further investigate TNFα’s role in muscle atrophy, we treated the murine myoblast cell line C2C12 with TNFα and induced differentiation of the cells. We found that TNFα significantly inhibited C2C12 cells myotube formation in a dose-dependent manner (Figure 1E). A similar inhibiting effect of TNFα on myotube formation was also found in primary myogenic cells from young C57BL/6 mice by magnetic-activated cell sorting (MACS) (Figure 1F), which is consistent with grip strength genetic association in population (Figure 1A **)**, as well as the development of muscle atrophy in denervated mice, associated with increased TNFα levels (Figures 1B–D).

### 3.3 Morroniside rescues TNFα-induced myotube atrophy *in vitro*

**FIGURE 2:**
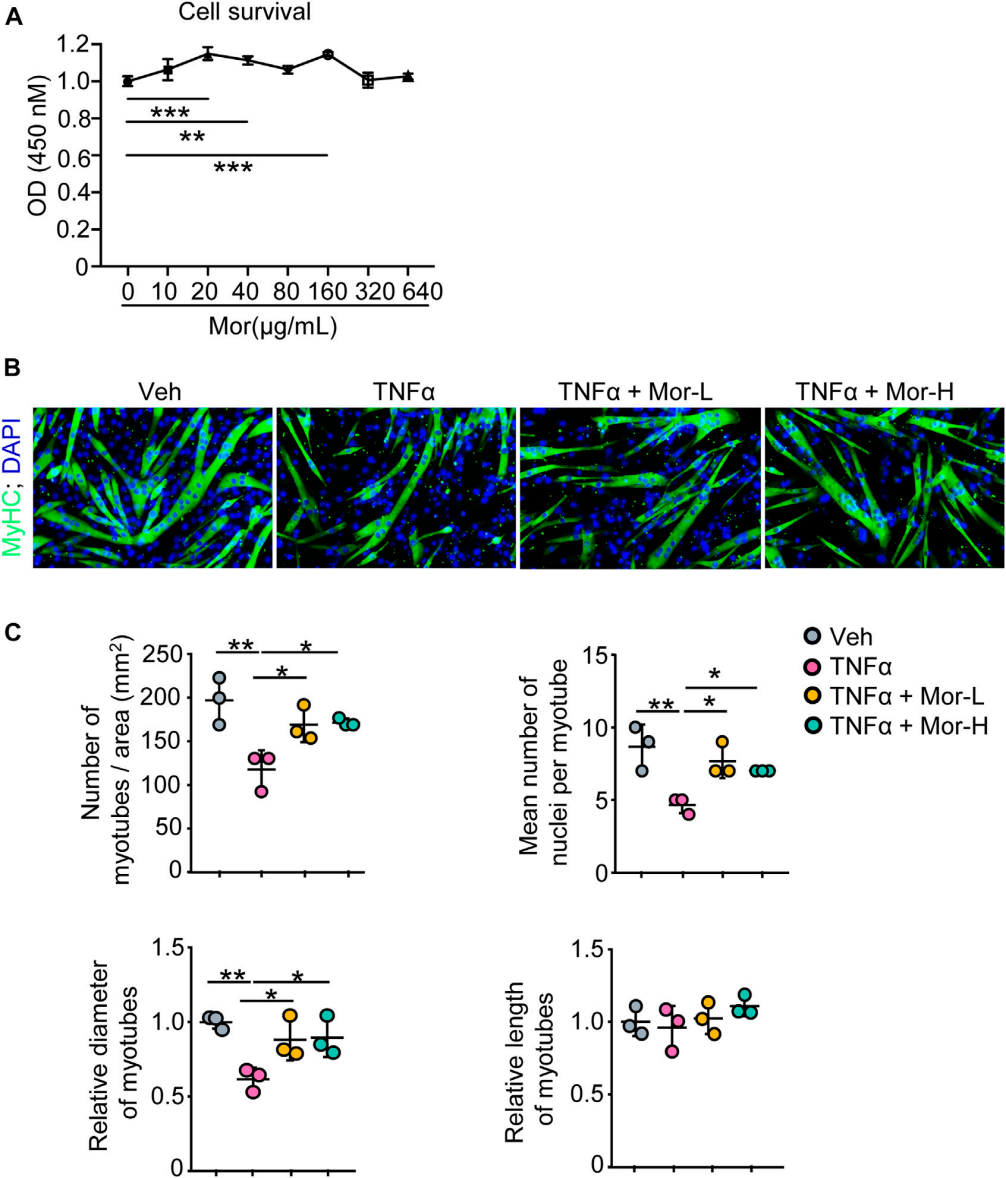
Morroniside rescues TNFα-induced myotube atrophy *in vitro*. **(A)** C2C12 cells were treated with indicated concentrations of morroniside (0–640 μg/ml) for 48 h, then the cell survival was examined by CCK8 kit. **(B–C)** C2C12 cells were treated with TNFα (10 ng/ml) and administrated with or without lower dose of morroniside (Mor-L, 80 μg/ml) or higher dose of morroniside (Mor-H,160 μg/ml), then the cells were induced to differentiation. **(B)** IF staining shows the effects of morroniside (Mor) on myotube formation from C2C12 cells treated with TNFα. **(C)** Number of myotubes/area, mean number of nuclei per myotube, relative diameter of myotubes, and relative length of myotubes were measured on IF images. n = 3 samples/group; *p < 0.05; **p < 0.01.

To find the potential process that TNFα is involved in and possible treatment for TNFα-induced muscle atrophy, we next performed pathway enrichment analysis with the KOBAS online database and found that TNFα is related to immune response, regulation of I-κB kinase/NF-κB signaling, and negative regulation of myosin-light-chain-phosphatase activity (Supplementary Table S4). Moreover, previously, we reported that morroniside can improve the progression of osteoarthritis by inhibiting canonical NF-κB p65/RelA signaling (Yu et al., 2021), suggesting morroniside could ameliorate TNFα-induced muscle atrophy. To investigate this possibility, here, we treated C2C12 cells with different doses of morroniside (0–640 μg/ml) *in vitro*, and found that 20–160 μg/ml morroniside could improve C2C12 cell survival, and there was no obvious toxicity on the cells when using up to 640 μg/ml morroniside (Figure 2A). Further, C2C12 cells were treated with TNFα and administrated with or without morroniside and the cells were induced to differentiation (Figure 2B). IF staining results showed that lower number of myotubes/area, mean number of nuclei per myotube, and relative diameter of myotubes were found in TNFα-treated C2C12 myotubes, compared to vehicle group, whereas these parameters from C2C12 myotubes treated with TNFα plus lower or higher dose of morroniside were similar to the values in vehicle group and significantly higher than those in TNFα-treated myotubes (Figures 2B, C). These data indicate that morroniside rescues TNFα-induced myotube atrophy *in vitro*.

### 3.4 Morroniside impedes skeletal muscle atrophy caused by denervation *in vivo*

**FIGURE 3:**
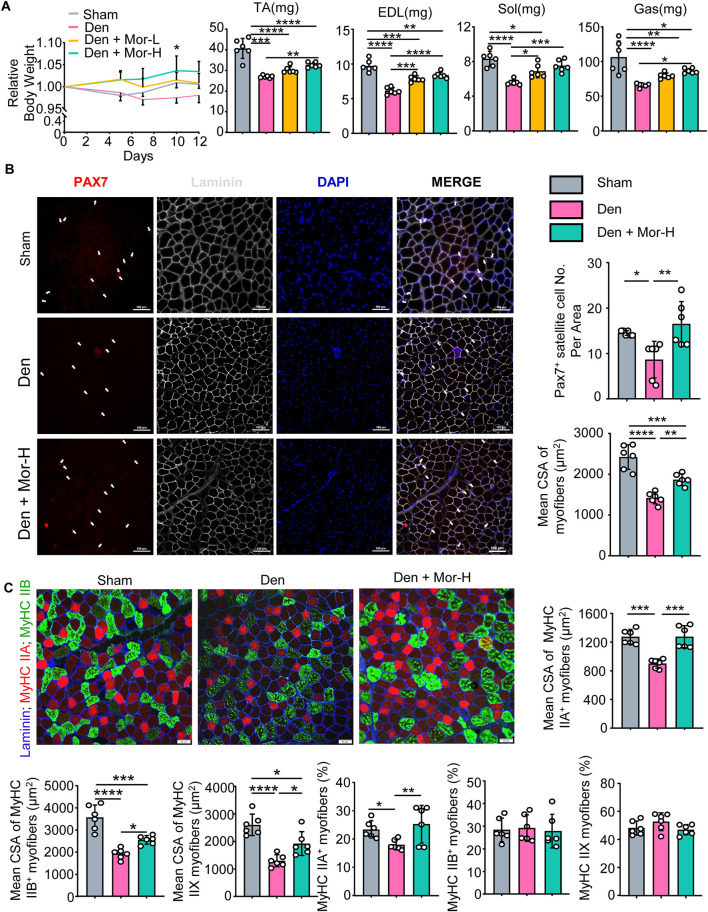
Morroniside impedes skeletal muscle atrophy caused by denervation *in vivo*. 2-month-old male C57BL/6 mice were randomly divided into Sham operation, Denervation (Den), Den + low-dose morroniside (Mor-L, 10 μg/kg), Den + high-dose morroniside (Mor-H, 20 μg/kg) groups based on body weights, and received sham operation or sciatic nerve transection and were treated with Veh or morroniside 1 x/day, starting 1 day after operation for 12 cycles. n = 6/group. **(A)** Relative body weight changes (*p < 0.05, Den + Mor-H vs. Den at d10) and the lean weights of the tibialis anterior (TA), extensor digitorum longus (EDL), soleus, and gastrocnemius (Gas) muscles in indicated groups. **(B–C)** Cryosections (10 μm thick) of TA muscles IF-stained for Pax7 (red), and laminin (gray) expression in **(B)** and Laminin (blue), MyHC IIA (red) and MyHC IIB (green) in **(C)**. **(B)** The numbers of Pax7^+^ satellite cells (white arrows), mean cross-sectional area (CSA) of myofibers and **(C)** Mean CSA of MyHC IIA^+^, IIB^+^, IIX (namely IIA^−^; IIB^−^) myofiber, the percentage of MyHC IIA^+^, IIB^+^ and IIX (namely IIA^−^; IIB^−^) myofibers were measured on IF images. n = 6 samples/group; *p < 0.05; **p < 0.01; ***p < 0.001; ****p < 0.0001.

To further investigate the effect of morroniside on denervation-induced muscle atrophy, sciatic nerve transection was performed on adult male C57BL/6 mice and the mice were randomly divided into sham operation, Denervation (Den), Den + low-dose morroniside, Den + high-dose morroniside groups. The body weight of denervated mice trended downwards over time, whereas low-dose morroniside tended to rescue body weight to the normal level and high-dose morroniside even significantly increased the body weight of mice subjected to denervation at day 10 post-surgery, compared with the denervation group (Figure 3A). Consistent with Figure 1D, the lean mass of tibialis anterior (TA), extensor digitorum longus (EDL), soleus, and gastrocnemius (Gas) muscles was decreased in the denervated mice, but was significantly increased in high-dose morroniside treated denervated mice (Figure 3A). The paired box transcription factor, Pax7, is an established muscle stem cell (MuSC) marker and plays a critical role in regulating MuSC proliferation (Li et al., 2020). Fiber type variety is related with functional diversity, changes in muscle fiber types influence contractile, metabolic and biochemical properties of the muscle (Qaisar et al., 2016). The exact type of muscle fibers impacted in TA after denervation, to the best of our knowledge, is to a great extent obscure. Of note, IF staining showed that the numbers of Pax7^+^ satellite cells (Figure 3B), mean cross-sectional area (CSA) of myofibers (Figure 3B), specifically, the mean CSA of MyHC IIA, IIB and IIX myofibers (Figure 3C), and the percentage of MyHC IIA myofibers (Figure 3C) in TA muscles from denervated mice was significantly lower than those from sham mice, whereas those from denervated mice treated with high-dose morroniside were similar to the values in sham mice and significantly higher than those in denervated mice (Figures 3B,C). There were no significant changes detected in the percentage of MyHC IIB and IIX fiber among the three groups (Figure 3C). Taken together, these data indicate that morroniside relieves skeletal muscle atrophy caused by denervation *in vivo*.

### 3.5 NF-κB signaling in response to TNFα is associated with grip strength in human

Our and other studies have shown that NF-κB signaling is involved in RA-, aging-, and denervation-related muscle atrophy in animal models (Li et al., 2020; Li et al., 2021b). To investigate if this signaling pathway in response to TNFα is associated with muscle atrophy and grip strength in human, we conducted a pathway-based genetic association study in population, and found that the weighted genetic risk score of NF-κB pathway in response to TNFα (including 198 genes) was significantly associated with grip strength, suggesting this TNFα-induced NF-κB pathway is related to muscle atrophy in human (Supplementary Table S3; Table 1). We further re-conducted the pathway-based weighted genetic risk score using the different threshold of *p*-values, and found that our finding remains significant (Table 1).

**TABLE 1:** The association of NF-κB pathway in response to TNF with right-hand grip strength.

The generation of weighted genetic risk score			Number of SNPs	*p*-value for pathway-based association
*p*-value threshold for included SNPs	*r* ^2^	Kb		
0.5	0.1	250	4453	<2e-16
0.4	0.1	250	3806	<2e-16
0.3	0.1	250	3045	<2e-16
0.2	0.1	250	2221	<2e-16
0.1	0.1	250	1274	<2e-16
0.05	0.1	250	746	<2e-16

Abbreviations: SNPs, single nucleotide polymorphisms.

### 3.6 Morroniside inhibits canonical and non-canonical NF-κB signaling in C2C12 myotubes treated with TNFα and muscles from denervated mice

**FIGURE 4:**
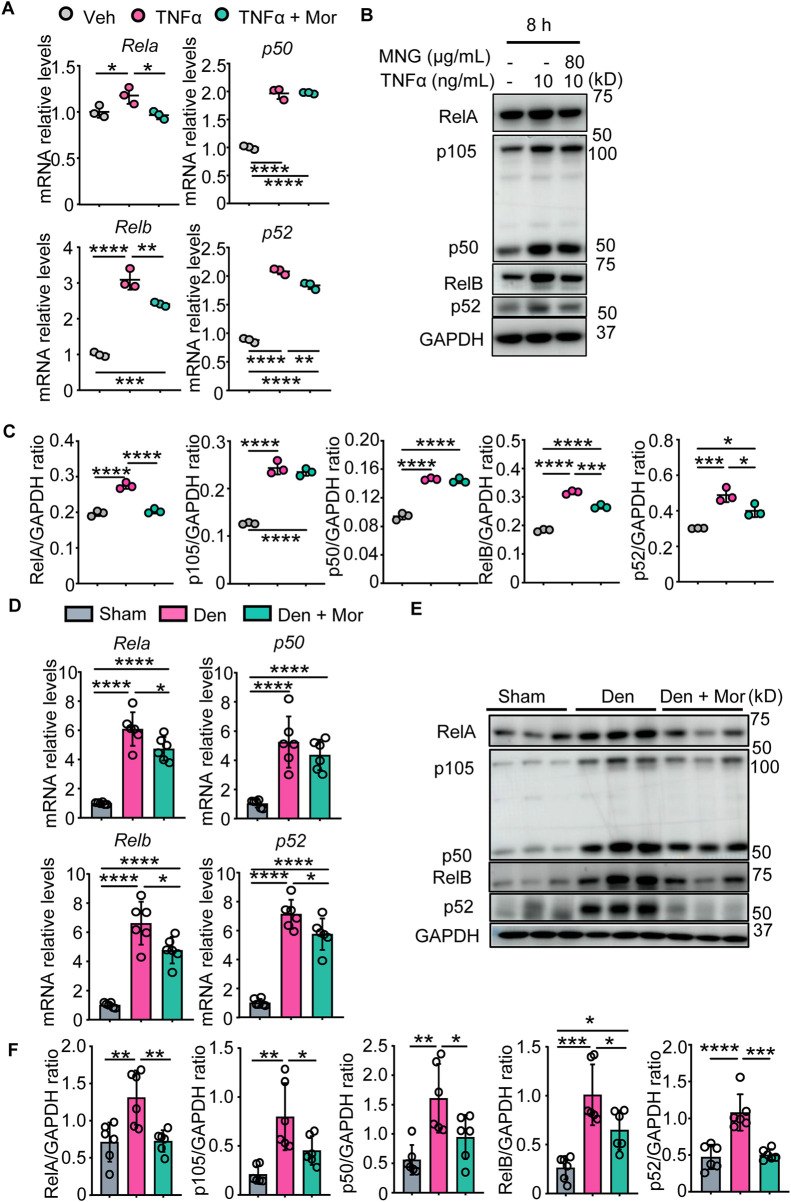
Morroniside inhibits canonical and non-canonical NF-κB signaling in C2C12 myotubes treated with TNFα and muscles from denervated mice. **(A–C)** C2C12 cells were treated with TNFα (10 ng/ml) and administrated with or without morroniside (Mor, 80 μg/ml), then the cells were induced to differentiation. **(A)** NF-κB mRNA and **(B)** protein levels in C2C12 myotubes were measured by qPCR and western blot, respectively. **(C)** Quantification of NF-κB protein levels in **(B)** by Image J. n = 3 samples/group. **(D–F)** 2-month-old male C57BL/6 mice were randomly divided into Sham operation, Denervation (Den), Den + morroniside (Mor, 20 μg/kg) groups based on body weights, and received sham operation or sciatic nerve transection and were treated with Veh or morroniside 1 x/day, starting 1 day after operation for 12 cycles. n = 6/group. **(D)** NF-κB mRNA and **(E)** protein levels in gastrocnemius muscles were measured by qPCR and western blot, respectively. **(F)** Quantification of NF-κB protein levels in **(D)** by Image J. n = 6 samples/group; *p < 0.05; **p < 0.01; ***p < 0.001; ****p < 0.0001.

Morroniside can inhibit canonical NF-κB p65/RelA in chondrocytes and colon tissues (Yuan et al., 2020; Yu et al., 2021), but its role on non-canonical NF-κB signaling remains unknown. To investigate if morroniside could inhibit canonical and non-canonical NF-κB signaling in muscle, we first treated C2C12 cells with TNFα or TNFα plus morroniside during differentiation. We found that TNFα stimulated transcription and translation of canonical NF-κB RelA, p50 and non-canonical NF-κB RelB, p52 genes in C2C12 myotubes (Figures 4A–C). Similar to this, denervation-induced mice had higher mRNA and protein levels of RelA, p50, RelB, and p52 in gastrocnemius muscles than sham mice (Figures 4D–F). Notably, morroniside treatment significantly reduced mRNA and protein levels of RelA, RelB, and p52, but not of p50, in C2C12 cells treated with TNFα, compared to TNFα treatment alone (Figures 4A–C). Moreover, morroniside-treated denervated mice had significantly lower mRNA levels of RelA, RelB, and p52, but not of p50, and lower protein levels of RelA, p50, RelB, and p52 in gastrocnemius muscles than denervated mice (Figures 4D–F).

### 3.7 Morroniside attenuates inflammatory mediators in C2C12 myotubes treated with TNFα and muscles from denervated mice

**FIGURE 5:**
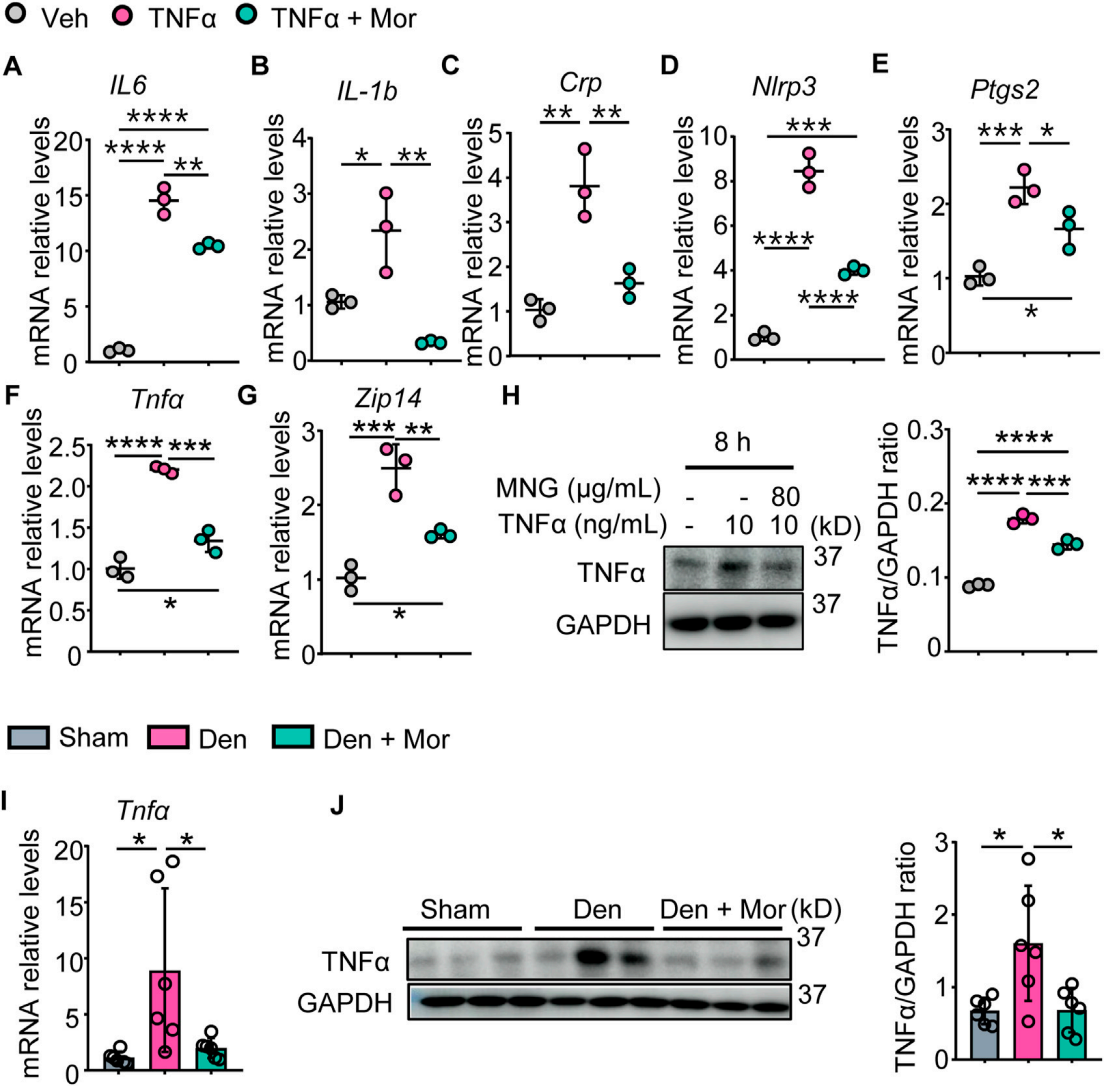
Morroniside attenuates inflammatory mediators in C2C12 myotubes treated with TNFα and muscles from denervated mice. **(A–H)** C2C12 cells were treated with TNFα (10 ng/ml) and administrated with or without morroniside (Mor, 80 μg/ml), then the cells were induced to differentiation. **(A–G)** mRNA levels of IL-6 **(A)**, IL-1b **(B)**, Crp **(C)**, Nlrp3 **(D)**, Ptgs2 **(E)**, Tnfα **(F)** and Zip14 **(G)** in C2C12 myotubes were measured by qPCR. n = 3 samples/group. **(H)** protein levels of TNFα in C2C12 myotubes were measured by Western Blots and were quantified by Image J. n = 3 samples/group. **(I–J)** 2-month-old male C57BL/6 mice were randomly divided into Sham operation, Denervation (Den), Den + morroniside (Mor, 20 μg/kg) groups based on body weights, and received sham operation or sciatic nerve transection and were treated with Veh or morroniside 1 x/day, starting 1 day after operation for 12 cycles. n = 6/group. **(I)** TNFα mRNA and **(J)** protein levels in gastrocnemius muscles were measured by qPCR and western blot, respectively. Quantification of TNFα protein levels by Image J. n = 6 samples/group; *p < 0.05; **p < 0.01; ***p < 0.001; ****p < 0.0001.

The anti-inflammatory effects of morroniside in muscle were examined, and results indicated TNFα increased IL-6, IL-1b, CRP, NLRP3, PTGS2 mRNA levels, as well as TNFα mRNA and protein levels in C2C12 myotubes (Figures 5A–H). Similarly, denervation-induced mice had higher mRNA and protein levels of TNFα in gastrocnemius muscles than sham mice (Figures 5I, J). Importantly, morroniside treatment significantly reduced mRNA and/or protein levels of those inflammatory factors in TNFα-treated C2C12 myotubes and gastrocnemius muscles from denervated mice (Figures 5A–J). The metal-ion transporter ZRT- and IRT-like protein 14 (ZIP14) is reported upregulated by TNFα and ZIP14-dependent zinc accumulation induces MyHC loss in cachexia (Wang et al., 2018). Of note, morroniside treatment significantly reduced ZIP14 mRNA levels in TNFα-treated C2C12 myotubes (Figure 5G).

### 3.8 Morroniside improves protein synthesis pathways in TNFα-treated myotubes

**FIGURE 6:**
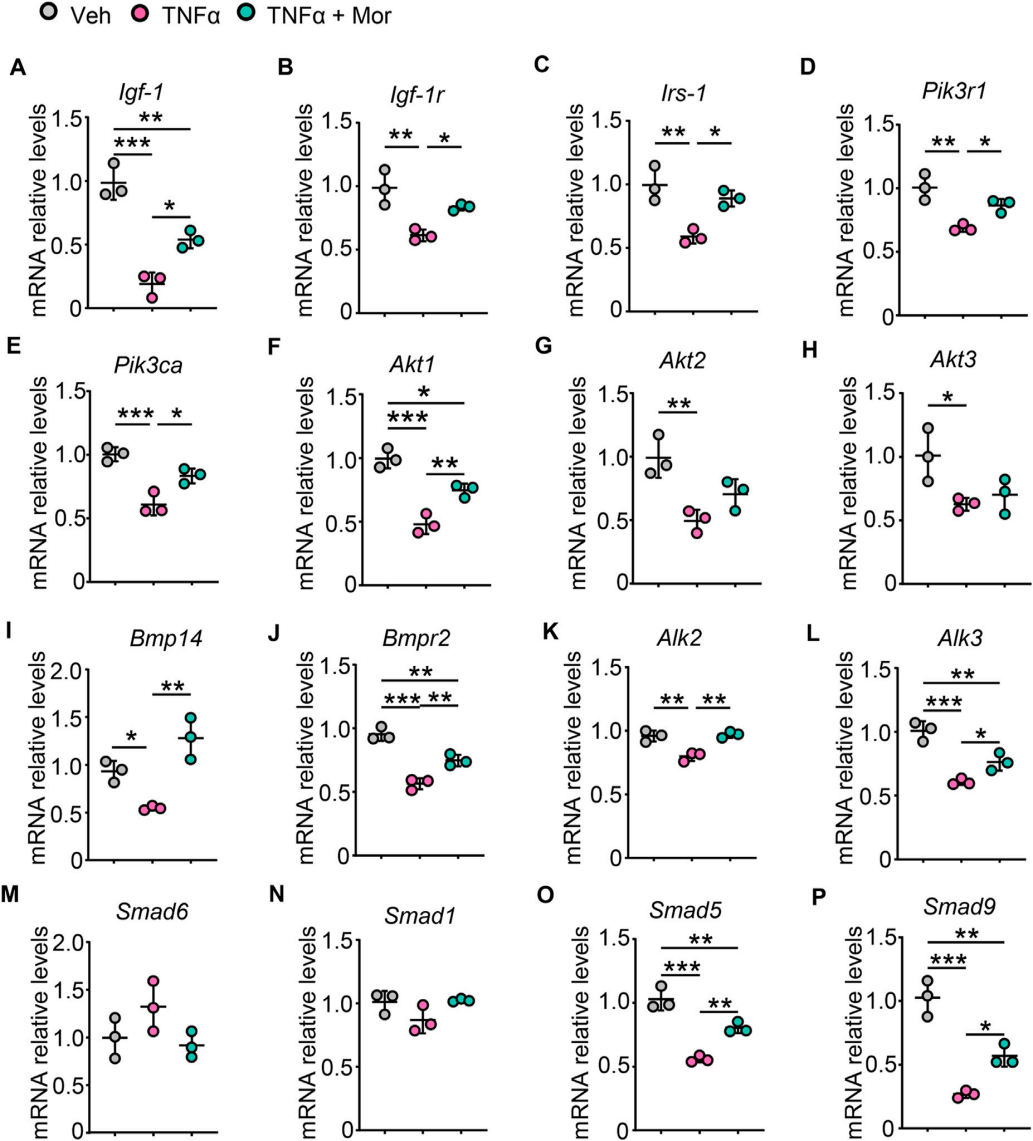
Morroniside improves protein synthesis pathways in TNFα-treated myotubes. **(A-P)** C2C12 cells were treated with TNFα (10 ng/ml) and administrated with or without morroniside (Mor, 80 μg/ml), then the cells were induced to differentiation. **(A-P)** mRNA levels of Igf-1 **(A)**, Igf-1r **(B)**, Irs-1 **(C)**, Pik3r1 **(D)**, Pik3ca **(E)**, Akt1 **(F)**, Akt2 **(G)**, Akt3 **(H)**, Bmp14 **(I)**, Bmpr2 **(J)**, Alk2 **(K)**, Alk3 **(L)**, Smad6 **(M)**, Smad1 **(N)**, Smad5 **(O)** and Smad9 **(P)** in C2C12 myotubes were measured by qPCR. n = 3 samples/group. *p < 0.05; **p < 0.01; ***p < 0.001.

We next investigated the effects of morroniside on protein synthesis pathways after TNFα treatment. TNFα reduced IGF-1 mRNA expression, which could be attenuated by morroniside treatment (Figure 6A). Also, morroniside attenuated TNFα-induced down-regulation on mRNA levels of IGF-1R (Figure 6B). Moreover, TNFα decreased the mRNA levels of IRS-1 adaptor protein (Figure 6C) and downstream PI3K regulatory subunit PIK3R1 (Figure 6D) and catalytic subunit PIK3CA (Figure 6E), as well as Akt1/2/3. Notably, morroniside up-regulated IRS-1, PIK3R1, PIK3CA and Akt1 in TNFα-treated C2C12 myotubes (Figures 6C–F). Besides, we also investigated another protein synthesis pathway, the BMP pathway. TNFα decreased mRNA expression of BMP ligand BMP14 (Figure 6I), BMP type II receptor BMPR2 (Figure 6J) and recruitment levels of type 1 receptors ALK2/3 (Figures 6K,L), which could be reversed by morroniside treatment (Figure 6I–L). Also, morroniside attenuated TNFα-induced increase of BMP pathway negative modulator Smad6 (Figure 6M) and TNFα-induced decrease of Smad1 (Figure 6N), although has no significance. Of note, morroniside significantly attenuated TNFα-induced decrease in the mRNA levels of Smad5/9 (Figures 6O,P). These results suggested that morroniside could protect C2C12 myotubes against TNFα-induced atrophy *via* promoting the IGF-1/IGF-1R/PI3K/Akt and BMP14/BMPR2/ALK2/3/Smad5/9 protein synthesis pathways.

### 3.9 Morroniside inhibits proteasomal and autophagic degradation pathways in C2C12 myotubes treated with TNFα and/or muscles from denervated mice

**FIGURE 7:**
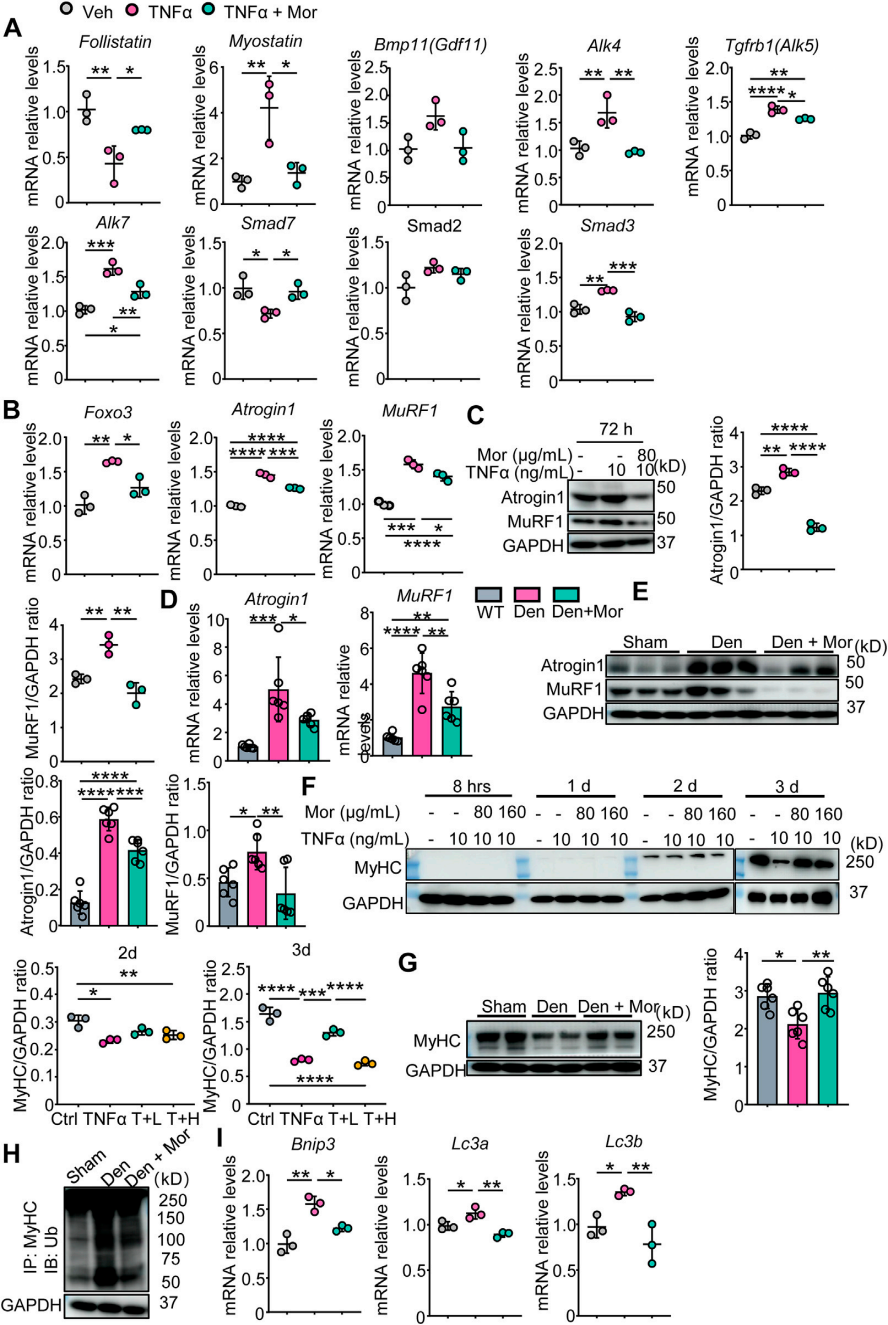
Morroniside inhibits proteasomal and autophagic degradation pathways in C2C12 myotubes treated with TNFα and/or muscles from denervated mice. **(A–C, F–J)** C2C12 cells were treated with TNFα (10 ng/ml) and administrated with or without morroniside (Mor, 80 or 160 μg/ml), then the cells were induced to differentiation. n = 3 samples/group. **(A–B)** Follistatin, Myostatin, Bmp11, Alk4, Alk5, Alk7, Smad7, Smad2, Smad3 **(A)**, Foxo3, Atrogin1 and MuRF1 **(B)** mRNA levels in C2C12 myotubes were measured by qPCR. **(C)** Atrogin1 and MuRF1 protein levels in C2C12 myotubes were measured by WB and quantified by Image J. n = 3 samples/group. **(D–E and G–I)** 2-month-old male C57BL/6 mice were randomly divided into Sham operation, Denervation (Den), Den + morroniside (Mor, 20 μg/kg) groups based on body weights, and received sham operation or sciatic nerve transection and were treated with Veh or morroniside (Mor) 1 x/day, starting 1 day after operation for 12 cycles. n = 6/group. **(D–E)** Atrogin1 and MuRF1 mRNA **(D)** and protein levels **(E)** in gastrocnemius muscles were measured by qPCR and WB, respectively. Quantification of Atrogin1 and MuRF1 protein levels by Image J. n = 6 samples/group. **(F)** MyHC protein levels in the cell lysates were measured by WB and quantified by Image J. T + L = TNFα +80 μg/ml Mor; T + H = TNFα +160 μg/ml Mor. n = 3 samples/group. **(G)** MyHC protein levels in the gastrocnemius lysates were measured by WB and quantified by ImageJ n = 6 samples/group, *p < 0.05; **p < 0.01. **(H)** WB of GAPDH and MyHC ubiquitination after IP with anti-MyHC antibody and IB with anti-Ub antibody in gastrocnemius lysates. **(I)** Bnip3, Lc3a, Lc3b mRNA levels in C2C12 myotubes were measured by qPCR. *p < 0.05; **p < 0.01; ***p < 0.001; ****p < 0.0001.

Next, the involvement of protein degradation signals was studied in morroniside’s protective mechanisms. First, we focused on myostatin protein degradation pathway. As shown in Figure 7A, TNFα significantly decreased the mRNA levels of myostatin pathway inhibitory cytokine Follistatin and negative modulator Smad7, increased ligand Myostatin, type I receptors ALK4/5/7 and effector Smad3 mRNA expression. Of note, these parameters were all reversed by morroniside in TNFα-treated C2C12 myotubes (Figure 7A). To investigate if morroniside inhibits FoxO3, Atrogin1, and MuRF1 expression and MyHC reduction in muscle, we performed qPCR and Western blots and found that in response to TNFα, FoxO3 mRNA levels and transcription and translation of Atrogin1 and MuRF1 were upregulated in C2C12 myotubes **(** Figures 7B,C). Similarly, denervated mice had higher Atrogin1 and MuRF1 mRNA and protein expression in gastrocnemius muscles than sham mice (Figure 7D,E). Remarkably, morroniside significantly reduced the mRNA and/or protein levels of these genes in TNFα-treated C2C12 myotubes and gastrocnemius muscles from denervated mice (Figures 7B–E). Consistent with this, MyHC protein levels in TNFα-treated C2C12 myotubes and gastrocnemius muscles from denervated mice were significantly lower than those in vehicle-treated myotubes and sham mice, whereas the values in TNFα plus morroniside-treated C2C12 myotubes and in gastrocnemius samples from denervated mice administrated with morroniside were largely similar to levels in vehicle-treated myotubes and sham mice, and significantly higher than those in TNFα-treated C2C12 myotubes and gastrocnemius muscles from denervated mice, respectively (Figures 7F,G). To further investigate the mechanism whereby morroniside prevented MyHC degradation, we measured the levels of MyHC ubiquitination and found that they were higher in gastrocnemius muscles from denervated mice than those from sham mice, but morroniside treatment significantly reduced the values than those from denervated mice (Figure 7H). We also investigated another protein degradation pathway, the autophagy-lysosome system. Our results showed that mRNA levels of Bnip3, LC3a, and LC3b, autophagy-related proteins, were significantly increased by TNFα (Figure 7I). Morroniside treatment markedly reduced TNFα-induced Bnip3, LC3a, and LC3b mRNA expression (Figure 7I). Accordingly, these data suggest morroniside blunted TNFα-induced myotubes atrophy and denervation-induced muscle atrophy through down-regulating myostatin pathway, ubiquitin-proteasome system and autophagy-lysosome system protein degradation pathways.

### 3.10 Morroniside has no overt side effects on liver and kidney in mice induced by denervation

**FIGURE 8:**
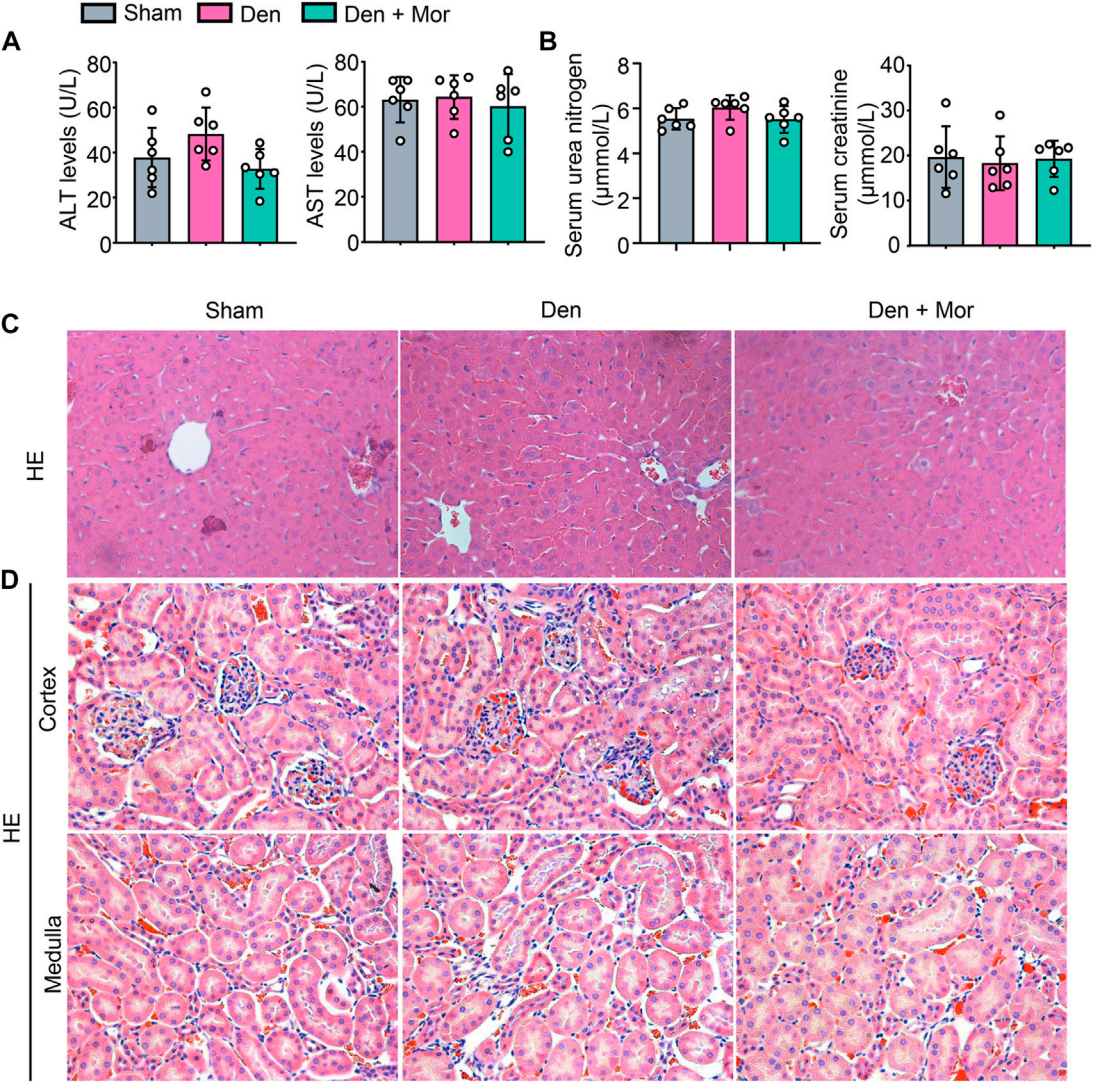
Morroniside has no overt side effects on liver and kidney in mice induced by denervation. **(A–D)** 2-month-old male C57BL/6 mice were randomly divided into Sham operation, Denervation (Den), Den + morroniside (Mor, 20 μg/kg) groups based on body weights, and received sham operation or sciatic nerve transection and were treated with Veh or morroniside (Mor) 1 x/day, starting 1 day after operation for 12 cycles. n = 6/group. **(A)** Serum ALT and AST levels. **(B)** Serum urea nitrogen and creatinine levels. **(C–D)** Represent images of HE staining for liver **(C)** and the cortex (upper panel) and medulla (lower panel) of kidney **(D)**.

Side effects of drugs prevent them from being used effectively (Tao et al., 2022). Although the efficacy of morroniside has been studied in different settings (Gao et al., 2021), its safety remains unknown. We further investigated if there were any adverse effects of morroniside in mice, and found that there was no significant difference in serum ALT and AST levels, two liver injury markers, among sham, Den and Den + Mor groups (Figure 8A). Similarly, morroniside also didn’t significantly change the serum urea nitrogen and creatinine levels, two renal injury markers, in denervated mice, compared to sham and denervation groups (Figure 8B). Moreover, the HE staining results indicated that morroniside didn’t induce any abnormal phenotype such as inflammatory infiltration, degeneration or necrosis of liver cells and renal epithelial cells in liver and kidney from denervated mice, compared with sham and denervation groups (Figure 8C). These data indicate that morroniside has no obvious side effects on liver and kidney in mice to treat denervation-induced muscle atrophy.

## 4 Discussion

**FIGURE 9:**
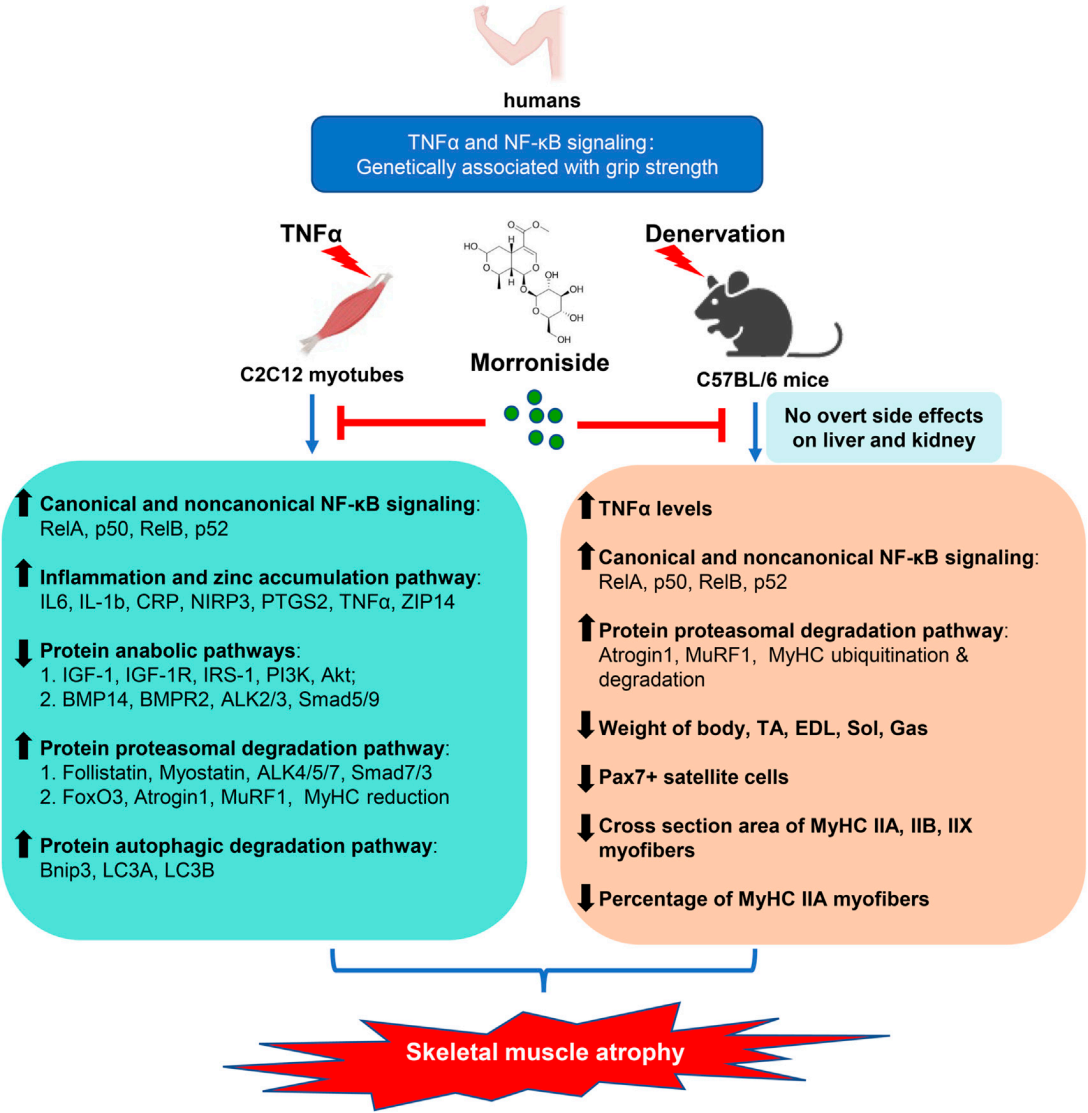
Proposed schematic diagram illustrating the protective efficacy, mechanisms and safety of morroniside against denervation-induced skeletal muscle atrophy. TNFα is elevated in muscle induced by denervation, activating NF-κB signaling, which is associated with atrophying muscle mass in mice and grip strength in population identified by genetic association studies. Morroniside attenuates TNFα-induced C2C12 myotubes atrophy *via* inhibiting canonical and non-canonical NF-κB signaling, inflammatory mediators and zinc accumulation pathway, proteasomal and autophagic protein degradation pathways, while improving protein synthesis pathway. In denervation-induced mice, morroniside decreases TNFα levels, canonical and non-canonical NF-κB signaling, proteasomal degradation pathway and increases weight of body, TA, EDL, Sol, Gas muscles, as well as Pax7+ satellite cells, cross section area of MyHC IIA, IIB, IIX myofibers and the percentage of MyHC IIA myofibers without obvious liver and kidney toxicity, therefore ameliorating muscle atrophy.

The present study reports a critical association between TNFα/NF-κB signaling and grip strength based on population genetic evidence, as well as the anti-atrophic efficacy and safety of morroniside in TNFα-treated C2C12 myotubes and/or inflammation-associated muscle atrophy induced by denervation. Morroniside-mediated anti-atrophy activity is related to inhibiting canonical and non-canonical NF-κB signaling, inflammatory mediators and zinc accumulation pathway, proteasomal and autophagic protein degradation pathways, while improving protein synthesis pathway (Figure 9). Therefore, we provide strong preliminary evidence for the anti-atrophic efficacy, mechanisms and safety of morroniside to support further drug development.

Skeletal muscle wasting occurs in a variety of pathogenic states ranging from inherited to acquired diseases, including Duchenne muscular dystrophy, Crohn’s disease, diabetes, aging, rheumatoid arthritis (RA) (Li et al., 2020), cachexia, cancer, disuse, and denervation (Thoma and Lightfoot, 2018), which are accompanied with the upregulation of pro-inflammatory cytokines in patients, such as TNFα, IL-6, and/or CRP. Our and others’ previous studies have indicated that NF-κB activation and downstream inflammatory cytokines (such as IL6, IL-1b, CRP, NIRP3, PTGS2, and TNFα) are key players in muscle atrophy in animal models (Thoma and Lightfoot, 2018; Li et al., 2020). The NF-κB signaling is activated *via* two distinct pathways: the canonical and non-canonical pathways. Canonical NF-κB signaling activation in satellite cells attenuates skeletal muscle regeneration following injury in adult mice (Straughn et al., 2019). Stimulation of non-canonical NF-κB impairs myogenic differentiation, muscle stem cell function and muscle regeneration in mice (Schmidt et al., 2021). GWAS has successfully identified a multitude of loci influencing the variability of different musculoskeletal traits, some of which subsequently have been experimentally verified (Trajanoska et al., 2019; Zhu et al., 2021). but to date, we are unaware of any genetic evidence on the association between TNFα/NF-κB pathway and grip strength in human. Here, based on data of 377,807 participants, we found 341 SNPs located within *Tnfα* gene region with genome-wide significant associations for grip strength, and revealed that NF-κB pathway (including 198 genes) in response to TNFα was significantly associated with grip strength (Figure 1A; Table 1), suggesting the important role of TNFα and NF-κB pathway in muscle atrophy in human. Indeed, we found TNFα levels were increased in atrophying muscle induced by denervation, which we showed inhibited myotube formation *in vitro* (Figures 1B–F). Morroniside has been suggested to inhibit canonical NF-κB p65/RelA signaling in different cells and tissues such as chondrocytes and colon tissues (Yuan et al., 2020; Yu et al., 2021). It is noteworthy that the present study further indicated that morroniside could attenuate both canonical (p65/RelA, p50) and non-canonical (RelB, p52) NF-κB signaling and downstream inflammatory factors (IL6, IL-1b, CRP, NIRP3, PTGS2, TNFα) expression in C2C12 myotubes treated with TNFα and/or muscle samples from denervated mice (Figure 4). Previously, we demonstrated that TNFα-expressing myeloid lineage cells, including macrophages and granulocytes, accumulate in skeletal muscles in mice during aging, which contributes to sarcopenia (Li et al., 2020), suggesting denervation-induced TNFα may also come from myeloid cells. In addition, redox imbalance may be involved because increased reactive oxygen species production is associated with denervation-induced skeletal muscle atrophy (Wang et al., 2021), which could activate NF-κB by stimulating inflammasomes (Forrester et al., 2018). However, further studies will be required to determine the TNFα source and potential changes in the intramuscular microenvironment during denervation.

The major skeletal muscle fiber types are type I, IIA, IIX, and IIB, according to presently dominating classification system for mammalian skeletal muscle based on MyHC isoforms (Westerblad et al., 2010). Type I fibers are slow-twitch fibers with predominantly oxidative metabolism. Type IIB and IIX fibers are fast-twitch fibers, mainly metabolizing glucose by glycolytic pathway. Type IIA fibers are intermediate fibers with fast speed of contraction but mixed (glycolytic/oxidative) metabolism. Because fiber type diversity is associated with functional diversity, alterations in muscle fiber types affect contractile, metabolic and biochemical properties of the muscle (Qaisar et al., 2016). Atrophy of both type I and type II fibers in soleus or EDL is typically observed in denervated muscles (Bobinac et al., 2000). The size of tibialis anterior (TA) muscles is also decreased during denervation (Reza et al., 2017). However, the exact type of muscle fibers affected in TA after denervation, to the best of our knowledge, is largely unknown. In this study, we found that not only the size of myofibers, specifically, type IIA, type IIB and type IIX fibers, in TA were all decreased, but also the percentage of type IIA fibers was reduced after denervation. Importantly, all these effects were attenuated by morroniside treatment (Figure 3 **)**. Interestingly, there is an opposite published result (Wan et al., 2020) that showed an increased proportion of type II muscle fibers in denervated TA muscles, claiming that slow-to-fast twitch muscle fiber conversion after denervation. However, previous studies have identified that TA muscle consists of IIB, IIX and IIA, but not of I fibers (Kammoun et al., 2014; Nakamichi et al., 2022), and we have also confirmed it by MyHC I IF staining (data not shown), reducing the likelihood of slow-to-fast fiber type shift in TA after denervation. Nevertheless, this study welcomes falsifiability.

Imbalance between protein synthesis and degradation causes skeletal muscle atrophy. IGF-1/IGF-1R/Akt and BMP pathways are two crucial skeletal muscle protein synthesis pathways. IGF1 is the potent anabolic factor that sustains muscle growth through binding and activating IGF-1R and downstream PI3K/Akt pathway (Sartori et al., 2021). BMP ligand BMP14 preferentially binds to type II receptors like BMPR2, before enhancing recruitment of type I receptors such as ALK2/3, which promote transcription factors Smad1/5/9(8) to form a complex with Smad4 to affect protein synthesis related transcriptional regulation (Sartori et al., 2021). inflammation inhibits IGF-1 levels and BMP pathway (Schakman et al., 2012; Daigang et al., 2016; Martin et al., 2021). Morroniside has been shown to activate PI3K/Akt/mTOR signaling in different types of cells, such as osteoblast precursors, granulosa cells, and human neuroblastoma cells (Li et al., 2021a; Deng et al., 2021; Liu et al., 2021). Notably, our study further indicated that morroniside attenuated the inhibition of both IGF-1/IGF-1R/PI3K/Akt signaling and BMP14/BMPR2/ALK2/3/Smad5/9 pathway induced by TNFα, which promote protein synthesis in C2C12 myotubes (Figure 6).

In addition, inflammation can induce muscle protein degradation through ubiquitin-proteasome system and autophagy-lysosome system (Schakman et al., 2012; Thoma and Lightfoot, 2018). NF-κB activation and downstream inflammatory mediators increase muscle-specific E3 ubiquitin ligases, Atrogin1 and MuRF1, which promote ubiquitin-proteasome degradation (Li et al., 2008; Huang et al., 2017; Li J. et al., 2020). Lipopolysaccharide (LPS)-induced FoxOs up-regulation activates ubiquitination-related genes, Atrogin1 and MuRF1, and autophagy-related genes, Bnip3, LC3A, LC3B (Mammucari et al., 2007; Schakman et al., 2012). In addition to inhibiting protein synthesis by inhibiting mTOR and Akt, Myostatin/activin pathway is also involved in protein degradation (Sartori et al., 2021). Myostatin/activin and GDF11 bind to type II receptors like ActRIIB/IIA and recruit type I receptors ALK4/5/7, which activate transcription factors Smad2/3 with Smad4 to promote ubiquitination-related protein degradation (Sartori et al., 2021). Moreover, TNFα induces myostatin activation *via* NF-𝜅B, which activates both ubiquitin-proteasome and autophagy-lysosome systems in myotubes *via* PI3k/Akt/FoxO3a signaling pathway (Wang et al., 2015). Morroniside is suggested to repress autophagy through PI3K/Akt/mTOR pathway in rat ovarian granulosa cells (Deng et al., 2021). In this study, we further indicated that morroniside attenuated the activation of Myostatin/activin pathway, FoxO3, and downstream genes/proteins related to ubiquitin-proteasome system and autophagy-lysosome system in C2C12 myotubes treated with TNFα or muscles from denervated mice (Figure 7). More importantly, mice after denervation challenge that received morroniside injection showed improvement in muscle weight, No. of pax7+ satellite cells, muscle cross-sectional area (MyHC IIA, IIB and IIX), and percentage of MyHC IIA + fibers (Figure 3). Collectively, the above results all presented a positive outcome by morroniside against inflammation-associated muscle atrophy induced by denervation.

Despite the great effort of scientists and pharmaceutical companies to identify effective drug targets and chemical compounds to counteract muscle loss, successful pharmacological treatments for atrophying muscle are absent in the clinic (Sartori et al., 2021). The possible reason is partly due to poor efficacy and/or serious side effects of some promising preclinical candidate drugs in clinical trials preventing them from being further developed (Linlin et al., 2021). For example, β2-adrenoreceptor agonists, clenbuterol or formoterol, can not only stimulate glycogen and lipids degradation, but also improve protein synthesis and prevent protein degradation by enhancing the PI3K/Akt/mTOR pathway and inhibiting FOXO transcriptional activation of the ubiquitin-proteosome and autophagy-lysosome pathways (Joassard et al., 2013). But, although effectively promoting muscle growth, their adverse effects—particularly short-term (tachycardia) and long-term (cardiac hypertrophy) cardiovascular risks—preclude their clinical use (Ryall and Lynch, 2008; Sartori et al., 2021). Various natural products, such as resveratrol, quercetin, ursolic acid, ecdysone, and vitamin D, have been reported to preserve or control skeletal muscle health (Qu et al., 2021). In particular, Epicatechin, a flavanol found in tea and other consumable plants, has also been demonstrated to enhance muscular function in some atrophy models by inhibiting FOXO1, Atrogin1, and MuRF1 (Li et al., 2020; Gonzalez-Ruiz et al., 2020). However, there is still debate on the role of natural products in skeletal muscle health (Qu et al., 2021). Although these natural substances have shown promise in preclinical research, it is yet unknown if they are clinically effective and safe (Otzel et al., 2021). In clinical studies, therapeutic interventions including etanercept (TNFα inhibitor) and neutralizing antibody infliximab that blocks TNFα had no success to treat muscle wasting induced by cachexia (Wiedenmann et al., 2008; Wu et al., 2013), while they have shown promising results in inflammatory muscle loss induced by RA and Crohn’s disease (Chen et al., 2013; Subramaniam et al., 2015). This conflict could be partly due to the fact that cachexia is a syndrome that is multifactorial in nature and any single therapy is insufficient to stop or prevent muscle loss (Zhou et al., 2016). Pharmacological studies have found that morroniside has a variety of biological activities such as anti-inflammatory, anti-oxidative, anti-apoptotic, anti-pyroptotic, anti-diabetic, neurotrophic, neuroprotective, and cardiovascular protective activities (Gao et al., 2021). However, there is no direct evidence indicating the curative potential of morroniside on skeletal muscle atrophy. Importantly, this study revealed the potent anti-inflammation and anti-atrophy of morroniside. Given that increased oxidative stress is associated with denervation-induced skeletal muscle atrophy (Wang et al., 2021), and the anti-oxidative and neurotrophic effects of morroniside, morroniside inhibited oxidative stress and repaired nerve, which further abrogated muscle atrophy is also possible. Thus, the multiple effects of morroniside could make it more effective than a single treatment such as etanercept or infliximab.

An appropriate positive control drug in study can show efficacy of the test treatment by showing it is as good as or better than the active control. However, according to The International Council for Harmonisation of Technical Requirements for Pharmaceuticals for Human Use (ICH) guidelines (https://www.ich.org/), in general, the positive drug should be recognized by the academic community in the relevant professional field, and has the most certain curative effect on the indications studied, and is the safest drug approved by the state for marketing, and especially is the drug listed in the latest pharmacopeia. So, it’s difficult to choose an appropriate active control due to the absence of definitely effective and safe and approved drugs in clinical for skeletal muscle atrophy. Besides, skeletal muscle atrophy is an inflammatory disease in general. we and others have demonstrated that morroniside poses anti-inflammation effects by inhibiting canonical NF-κB p65/RelA signaling in different cells and tissues such as chondrocytes and colon tissues (Yuan et al., 2020; Yu et al., 2021). So, we didn’t set a positive drug group in this study. However, we recognize this limitation and an appropriate promising active control reported in preclinical and/or clinical studies will be chosen to test the superiority of morroniside in future preclinical studies.

Morroniside is the main component with medicinal properties of *C. officinalis*, which is an herb and food plant in east Asia, and its therapeutic polypharmacology has been clinically used in long-standing history according to the theory of TCM (Gao et al., 2021). Many TCMs are consumed as dietary supplements on a daily basis as having fewer side effects, which might lead to better compliance in patients with chronic diseases (Wu et al., 2022). It is noteworthy that we did not observe any adverse reaction or notable change in appearance, behavior or diet of the mice upon morroniside treatment. Moreover, no signs of redness and swelling or edema in any part of the body were seen. no significant toxicity or pathology was found in any organs including liver and kidney (Figure 8). Therefore, morroniside has no obvious adverse effects in mice. However, further randomized controlled trials are warranted to evaluate whether morroniside can be a potentially safe and effective drug for the treatment of muscle atrophy in human.

In summary, the present study is the first to combine the population, cells and animal evidence to demonstrate the anti-atrophic effect of morroniside *in vitro* and *in vivo* models of inflammation-related muscle atrophy. Morroniside counteracts inflammation-related muscle atrophy, at least in part, *via* inhibiting canonical and non-canonical NF-κB signaling, inflammatory factors, and regulating protein anabolic/degradation.

## Data Availability

The datasets presented in this study can be found in online repositories. The names of the repository/repositories and accession number(s) can be found in the article/Supplementary Material.
